# External and Internal Loads of Transition Games Are Affected by the Presence of an Opposing Team and Their Initial Positions

**DOI:** 10.5114/jhk/191850

**Published:** 2024-12-19

**Authors:** Jose A. Asian-Clemente, Iván Asín-Izquierdo, Bernardo Requena, Carlos Galiano

**Affiliations:** 1 Football Science Institute, FSI Lab, Granada, Spain.; 2 Physical Performance and Sports Research Center, Department of Sports and Computer Sciences, Faculty of Sport Sciences, Pablo de Olavide University, Seville, Spain.; 3 Department of Musical, Plastic and Corporal Expression, Faculty of Social and Human Sciences, University of Zaragoza, Teruel, Spain.; 4 Department of Communication and Education, Universidad Loyola Andalucía, Seville, Spain.

**Keywords:** time-motion, test, soccer, high-speed running, counterattack

## Abstract

The objectives of this study were to compare the external and internal loads of transition games (TGs) without the opposition and TGs with different types of the opposition, and to assess their impact on a series of speed and strength tests. The external and internal loads of 18 young professional players (age: 14.79 ± 0.18 years; body height: 171.9 ± 6.5 cm; body mass: 62.1 ± 7.5 kg) were monitored in three TGs: 1 vs. 0 (no opposition; striker vs. goalkeeper), 1 vs. 1_Front_ (defender between the goal and the striker), and 1 vs. 1_Behind_ (defender behind the striker). Peak velocity (PeakV), distance covered (DC) above 24.0 km•h^−1^, 21.0–23.9 km•h^−1^, 18.0–20.9 km•h^−1^, 13.0–17.9 km•h^−1^, accelerations and decelerations above 2.5 m•s^−2^ and below −2.5 m•s^−2^ as well as the rate of perceived exertion (RPE) were obtained. Before and after each TG, vertical jump performance and sprint ability were assessed. PeakV and DC ≥24 km•h^−1^ differed among drills, showing superior outcomes in the 1 vs. 1_Behind_ format. DC 21–23.9 km•h^−1^ showed significant differences among drills, with larger distances covered in the 1 vs. 0 format. The number of accelerations-decelerations was significantly different among drills. RPE response differed among drills, with larger values for 1 vs. 1_Behind_ and 1 vs. 1_Front_ formats. All post tests showed an effect on time response without significant group interaction. The 1 vs. 1_Behind_ and 1 vs. 0 formats induce higher values for high speed, sprint and accelerations than the 1 vs. 1_Front_ format, which shows better performance in decelerations.

## Introduction

Load monitoring is considered crucial in soccer for accurate training prescription and evaluation, which subsequently leads to potential improvements in physical fitness (Foster et al., 2001) and better adaptation to soccer competitions. In recent years, soccer coaches, technical staff and sports scientists have placed a significant emphasis on monitoring training and match loads (Miguel et al., 2021). The process of continuous monitoring leads to greater insights into the actual training load (Jaspers et al., 2017; Silva et al., 2023), making it one of the most important aspects for technical staff. The importance of training load monitoring is such that it has now become a key element for performance optimization and injury reduction in professional soccer (Jaspers et al., 2017).

The study of training loads has received significant attention in the scientific literature (Hader et al., 2019; Miguel et al., 2021; Rabano-Muñoz et al., 2023; Silva et al., 2018), with particular relevance attributed to works describing the training load of specific soccer drills (Chena et al., 2022; Clemente et al., 2023; Clemente and Sarmento, 2021; Dello-Iacono et al., 2023). Most soccer drills analyzed in the literature are designed for the entire squad or a large number of players. However, it is now understood that there are specific contexts where individual training sessions or smaller groups of players are necessary, with injured and non-starting players being the most common cases. These specific contexts need special attention; for example, injured players, during the rehabilitation process, need to be exposed to appropriate loads to enhance their performance following an adequate progression of loads in the return to play process (Gabbett, 2016). Similarly, the status of non-starting players should also be considered, as their reduced weekly physical training, combined with a lower match load, could have an impact on their long-term fitness levels (Paraskevas and Hadjicharalambous, 2018). Since it has been proposed that match participation can help maintain or even improve players’ fitness (Silva et al., 2011; Sporis et al., 2011), injured and non-starting players should seek suitable training stimuli through specific drills with a reduced number of participants. This is particularly crucial when their accumulated chronic load is low due to reduced match and training loads, but they are subjected to a high acute load, such as participating in a full match or being exposed to intense training stimuli. Under such conditions, athletes may experience high fatigue, increasing the risk of injury (Gabbett, 2016).

Taking into account that an insufficient training load does not induce functional adaptation, and an excessive training load may lead to an increased risk of injury (Gabbett, 2016), soccer coaches need to design tasks that address the aforementioned particularities. Recently, a new drill called transition games (TGs) has emerged (Asian-Clemente et al., 2022, 2023a, 2023b), which could be suitable for this objective. TGs are defined as highly demanding tasks to stimulate high-intensity effort, in which players have to attack and defend counterattack situations and rapid attacks, combined with goal shooting (Asian-Clemente et al., 2023a, 2023b). These tasks have demonstrated higher demands in terms of high accelerations and moderate to very high-intensity running compared to large-sided games and official matches (Asian-Clemente et al., 2022, 2024). This aspect could be of interest for the training load management of injured and non-starting players. Additionally, these games do not require a large number of players, as has been demonstrated in a previous study that involved only two players (Asian-Clemente et al., 2023b), which aligns with the numerical situations in the special contexts described above.

Another important consideration is that soccer coaches can use TGs in various shapes and formats, combining technical-tactical and physical aspects to train scoring opportunities in counter-attack actions. Often, they adjust defensive positioning to enhance players' abilities in these situations. Given that these training tasks undergo changes in their load orientation based on constraint manipulation (Asian-Clemente et al., 2023a, 2023b), it would be necessary to study the effects of these modifications on players’ loads and fatigue during TGs with a reduced number of participants (1 or 2 players), which could be easily incorporated for reconditioning injured players or non-starting players. For these reasons, the objectives of this study were: 1) to compare the external and internal loads of a TG without the opposition and two TGs with different types of the opposition, and 2) to assess their impact on a series of speed and strength tests.

## Methods

### Participants

Eighteen young soccer players (age: 14.79 ± 0.18 years; body height: 171.99 ± 6.56 cm; body mass: 62.07 ± 7.45 kg) from a professional academy of a Spanish first division club participated in this study. Players participated in five training sessions (80–120-min duration) and one competitive match (normally Sunday) per week. The data employed for conducting this study were acquired from daily monitoring of workloads during the team’s training, in which players’ activities were measured over the competitive season (Winter and Maughan, 2009), thus ethics committee clearance was not required. Nevertheless, the study followed the recommendations of the Declaration of Helsinki, and participants were informed of the study’s design and aims, giving their consent before it started.

### Measures

External and internal loads were monitored using a GPS system (Kinexon GNSS, Precision Technologies, Munich, Germany). Peak velocity (PeakV), distance covered at above 24.0 km•h^−1^ (DC ≥24 km•h^−1^), distance covered between 21.0 and 23.9 km•h^−1^ (DC 21–23.9 km•h^−1^), distance covered between 18.0 and 20.9 km•h^-1^ (DC 18–20.9 km•h^−1^), distance covered between 13.0 and 17.9 km•h^−1^ (DC 13–17.9 km•h^−1^), accelerations and decelerations above 2.5 m•s^−2^ and below −2.5 m•s^−2^ and the RPE were recorded to quantify the load of the TGs. These variables have been used previously in the literature to describe training loads (Asian-Clemente et al., 2021, 2023a, 2023b; Gualtieri et al., 2023).

### Design and Procedures

**Figure 1:**
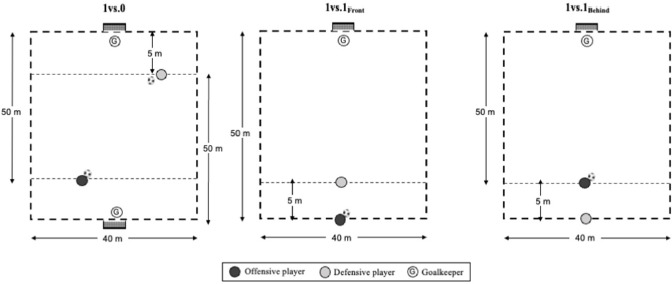
Graphical representation of TGs.

**Figure 2:**
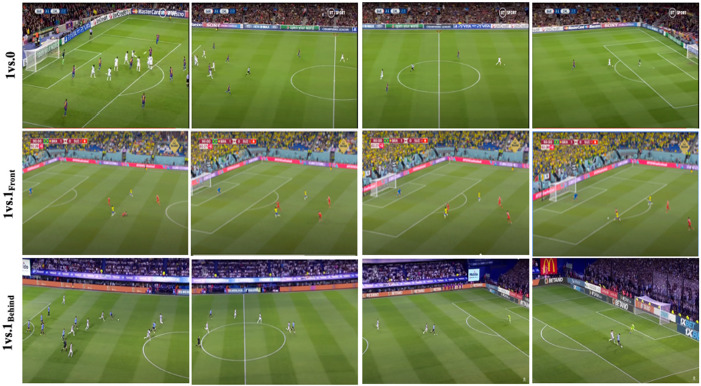
Examples of tasks in competition matches.

Data were collected during the latter part of the 2022–2023 season, when training loads of TGs were monitored over a span of 6 weeks. These specific TGs were selected by two highly experienced professional coaches with expertise in analyzing different match situations, particularly focused on counter-attacks and transitions in soccer matches. TGs under scrutiny are illustrated in Figure 1. Building on prior research (Asian-Clemente et al., 2022, 2023a, 2023b, 2024), two teams, each comprising nine players, engaged in transition plays to score goals across three realistic scenarios of counter-attacks (Figure 2). Every week, soccer players took part in a distinct TG, each of which was repeated twice. These TGs presented varying levels of the opposition. One was structured solely with the opposition of a goalkeeper (1 vs. 0), while the others involved both a goalkeeper and one defender, who adjusted their starting positions: either in front of (1 vs. 1_Front_) or behind (1 vs. 1_Behind_) the attackers. Regardless of the TG's format, the objective remained consistent: offensive players aimed to attack the opponents' goal to score, while defenders and goalkeepers tried to prevent them from scoring.

The tasks commenced upon the coach's signal. Following the initial movement, if the goal was achieved or the play concluded (due to the goalkeeper's save or the ball going out of play), all players rested while new teammates initiated the game. However, during the 1 vs. 1 scenario, if defensive players intercepted the ball, they were required to swiftly launch an attack towards the opposing goal. Regardless of the outcome of the subsequent play, participants were instructed to rest, and a new set of teammates would start after a new signal from the coach. Similarly, in any of the aforementioned instances, if players in possession of the ball failed to execute a rapid counter-attack, resulting in a positional play, coaches would terminate the play, allowing new players to take over. This sequence repeated alternately between the teams throughout the entire duration of the task. Initially, teams were assigned roles (offensive or defensive), and upon the participation of all players, these roles were exchanged. In the 1 vs. 0 format, both teams attacked different goals, and players commenced each attack following the coach's signal.

These TGs consisted of two sets lasting 8 min each, separated by 2 min of passive recovery. In cases where some players had not completed the same number of repetitions as their teammates when the time elapsed, the drill continued until all participants reached an equal number of repetitions. In all three TG formats, players were sorted into well-balanced teams based on technical and tactical proficiency, competitive experience, player positions, and subjective evaluations made by coaches. The team's goalkeepers also took part in the activities, but were not directly monitored. Coaches introduced additional balls as required to prevent any disruption in play, and they verbally encouraged players to maintain a high work rate. Soccer players in this study were already well-acquainted with these games as they had been repeated multiple times during the season. These drills were frequently used across all academy teams.

Before and after TGs, strength and speed abilities of soccer players were assessed using jump and sprint tests. The protocol employed had previously been used in a similar study (Asian-Clemente et al., 2023b). Prior to taking these measurements, all participants completed a standardized 15-min warm-up, consisting of 5 min of low-intensity running, 5 min of mobility and active stretching, and 2 sets of incremental 30-m running sprints.

The players' jumping ability was assessed using a vertical countermovement jump (CMJ) conducted before and after each measured soccer drill, employing an infrared timing system (Optojump, Microgate, Bolzano, Italy). The best result from three CMJs was selected for analysis. Each CMJ attempt was separated by a 45-s passive recovery period before the subsequent jump. Once three valid results were achieved, players were provided with a 2-min rest interval to prevent potential fatigue effects before the sprint test. For the 30-m standing-start all-out sprint performed using photocell gates (Witty, Microgate, Bolzano, Italy), times were recorded at both the 30-m (T30) and the 15-m mark (T15) for analysis. Coaches provided verbal encouragement during all sprints to ensure maximal players’ performance.

Following the speed test, TGs commenced with players who had completed the test first. Participants were well-acquainted with these tests as they were regularly conducted during routine academy testing. Four experienced researchers supervised the intervention to ensure accurate test execution and collected the rate of perceived exertion (RPE) values using the Borg CR10-scale (Inoue et al., 2022). RPE values were individually recorded before and immediately after the completion of each drill. Players were educated and accustomed to using this scale, as it was routinely employed by their coaches. This intervention took place on Wednesday (Match Day − 4, following a day off), at the initial part of the session. After the intervention, players continued the training session with possession games and a training match.

### Statistical Analysis

Data were presented as mean ± standard deviation. The Shapiro-Wilk test confirmed the normality assumption for the data and one way repeated measures ANOVAs determined the effect of drills (1 vs. 0, 1 vs. 1_Behind_ and 1 vs. 1_Front_) on the locomotor, mechanical and internal variables studied. Two-way repeated measures ANOVAs were performed to determine the effect of time (pre-post) and the drill on the CMJ, T30 and T15 as well as the interaction between the drill (group) and time. Partial eta-squared (η^2^_p_) was calculated as a measure of effect size, interpreted as trivial < 0.01, 0.01 ≤ small < 0.06, 0.06 ≤ moderate ≤ 0.15 and large ≥ 0.15 (Cohen, 2013). For all statistical analyses, a significance level of *p* < 0.05 was established. Bonferroni post-hoc corrections for multiple comparisons were employed when significant effects were reported within the ANOVA. Effect size and 95% confidence intervals were used to evaluate the magnitude of differences, interpreted as < 0.2 = trivial; 0.2–0.6 = small; 0.6–1.2 = moderate; 1.2–2.0 = large; > 2.0 = very large (Cohen, 1977). The above statistical analyses were performed using JASP software (JASP Team 2019, Version 0.11.1, University of Amsterdam).

## Results

Table 1 shows the descriptive analysis of the locomotor, mechanical and internal responses during the three different transition drills. Every locomotor and mechanical variable analyzed showed statistical differences depending on the presence and placement of a defender in the transition drill. PeakV statistically differed between groups (F = 10.80; η^2^_p_ = 0.207; *p* < 0.001; large) showing the fastest speeds for the 1 vs. 1_Behind_ format. The distance covered ≥24 km•h^−1^ (F = 63.83; η^2^_p_ = 0.606; *p* < 0.001; large) also showed the largest values for the 1 vs. 1_Behind_ format. DC 21–23.9 km•h^−1^ showed significant differences between drills (F = 28.26; η^2^_p_ = 0.405; *p* < 0.001: large) with larger distances covered for the 1 vs. 0 format. DC 18–20.9 km•h^−1^ (F = 9.58; η^2^_p_ = 0.187; *p* < 0.001; large) showed superior responses for 1 vs. 1_Front_ and 1 vs. 0 formats, while for DC 13–17.9 km•h^−1^ (F = 66.31; η^2^_p_ = 0.615; *p* < 0.001; large), the 1 vs. 1_Front_ format showed the largest outcomes (Figure 1). The number of accelerations was similar (F = 118.23; η^2^_p_ = 0.740; *p* < 0.001; large) for 1 vs. 0 and 1 vs. 1_Behind_ formats and significantly greater in comparison with the 1 vs. 1_Front_ format (Figure 1). The 1 vs. 1_Front_ format showed significantly superior deceleration outcomes (F = 23.97; η^2^_p_ = 0.366; *p* < 0.001; large) in comparison to 1 vs. 0 and 1 vs. 1_Behind_ formats (Figure 1). The RPE showed significant differences between drills (F = 8.93; η^2^_p_ = 0.177; *p* < 0.001; large) with larger values for 1 vs. 1_Behind_ and 1 vs. 1_Front_ formats.

**Table 1:** Descriptive data of locomotor, mechanical and internal response to the three transition games.

	1 vs. 0	1 vs. 1_Front_	1 vs. 1_Behind_	Group effect	
				η^2^_p_	*p*
**Max. Speed (km•h^−1^)**	28.48 ± 1.26	29.03 ± 2.39	30.04 ± 1.17	0.207	< 0.001
**DC ≥24 km•h^−1^ (m)**	188.85 ± 116.33	117.93 ± 60.60	351.72 ± 56.38	0.606	< 0.001
**DC 21–23.9 km•h^−1^ (m)**	172.59 ± 58.63	98.60 ± 35.99	105.38 ± 18.92	0.405	< 0.001
**DC 18–20.9 km•h^−1^ (m)**	100.48 ± 24.66	103.73 ± 30.68	77.59 ± 16.88	0.187	< 0.001
**DC 13–17.9 km•h^−1^ (m)**	149.74 ± 42.68	195.27 ± 32.43	98.21 ± 18.05	0.615	< 0.001
**Accelerations**	14.93 ± 2.77	6.23 ± 2.96	15.21 ± 1.72	0.740	< 0.001
**Decelerations**	1.70 ± 1.59	6.43 ± 3.10	4.07 ± 2.71	0.366	< 0.001
**RPE**	6.11 ± 1.25	7.00 ± 0.91	7.13 ± 0.74	0.177	< 0.001

**Notes**: Data shows Mean ± SD. Max Speed: maximal speed attained during the task; DC: distance covered; Accelerations and Decelerations are presented with the number of actions performed above 2.5 m•s^-2^ and below -2.5 m•s^-2^; RPE: Rate of Perceived Exertion

Table 2 and Figure 4 show the descriptive analysis of fatigue mechanical response after each transition drill. Lower limb mechanical response did not show any time*group interaction (F < 2.55; η^2^_p_ < 0.058; *p* > 0.084; small to trivial). All post tests showed an effect on time response (F > 10.30; η^2^_p_ > 0.110; *p* < 0.002; moderate to large).

**Table 2:** Descriptive and statistical analysis of the mechanical fatigue after the three transition games.

	1vs.0		1vs.1_Front_		1vs.1_Behind_		Time effect			Time * group interaction	
	Pre	Post	Pre	Post	Pre	Post	η^2^_p_	*p*	η^2^_p_		*p*
**T15 (s)**	2.48 ± 0.41	2.50 ± 0.13	2.37 ± 0.06	2.40 ± 0.07	2.45 ± 0.11	2.48 ± 0.10	0.110	0.002	0.009		0.701
**T30 (s)**	4.37 ± 0.20	4.41 ± 0.23	4.18 ± 0.11	4.26 ± 0.12	4.29 ± 0.18	4.38 ± 0.16	0.218	< 0.001	0.021		0.413
**CMJ (cm)**	38.36 ± 4.72	37.29 ± 5.29	38.22 ± 5.11	36.96 ± 4.71	37.11 ± 4.68	36.82 ± 5.12	0.209	< 0.001	0.058		0.084

**Notes**: Mean ± SD. T15: time for the 15-m sprint line test; T30: time for the 30-m sprint line test; CMJ: counter-movement jump

**Figure 3:**
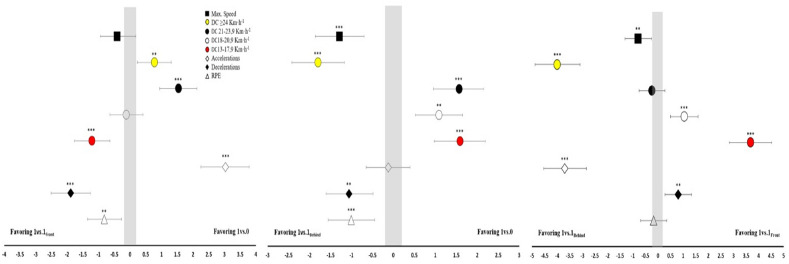
Comparison of external and internal loads of TG analysed. **Notes**: Difference scores (95% confidence intervals) among the three transition drills. Max Speed: Maximal speed attained during the drill; DC: Distance Covered; Accelerations and Decelerations are presented as the number of actions performed above 2.5 m•s^−2^ and below −2.5 m•s^−2^; RPE: Rate of Perceived Exertion; p-values are indicated as: *: p < 0.05; **: p < 0.01 and ***: p < 0.001

**Figure 4:**
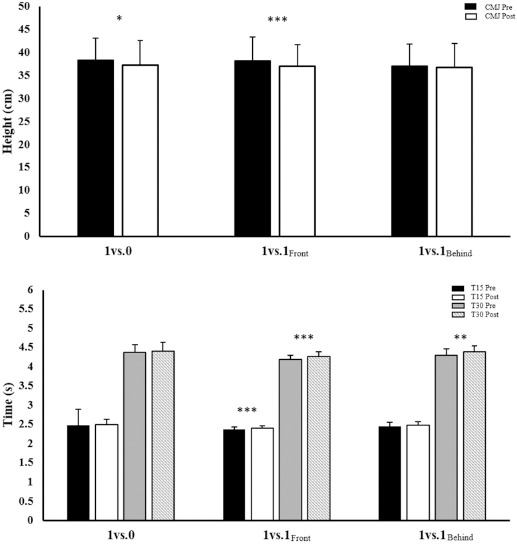
Comparison of test results pre- and post-TGs analysed. **Notes**: Changes in CMJ height and sprint time prior and after each transition game. Data are mean ± SD. T15: time for the 15-m sprint line test; T30: time for the 30-m sprint line test; CMJ: Counter-Movement Jump; p-values are indicated as: *: p < 0.05; **: p < 0.01 and ***: p < 0.001

## Discussion

This study was based on the analysis of internal and external loads in three different TGs to provide information on the two main objectives of the study: 1) to compare the external and internal loads of a TG without the opposition and two TGs with different types of the opposition, and 2) to assess the levels of acute induced fatigue on a series of speed and strength tests. The three training tasks showed clear differences in the internal-external load variables, thus each task provided a different context. However, fatigue induced in the tests performed seemed to be quite similar.

From a locomotor and mechanical standpoint, our results showed that TGs studied produced a high load in high-intensity and speed variables, both in peak velocity, and distance by speed bands (Table 1). Nevertheless, due to the intrinsic characteristics of each task, each TG showed a tendency for a different variable, which may be an important consideration when programming and designing appropriate training. Since the length of the field was relatively high for the number of players, PeakV was high in the three TGs (>28.48 km•h^−1^). In fact, velocities in the T30 test were lower than those achieved in TGs, which indicates that longer than 30-m linear sprints are necessary to measure maximum sprint capacity in soccer players (Buchheit et al., 2012). The TGs studied seemed a suitable strategy to develop speeds close to maximum competition speeds as opposed to other types of tasks, especially in counter-attack contexts (Asian-Clemente et al., 2024). In this sense, the 1vs.1_Behind_ format produced the largest value in PeakV (ES > 0.79; *p* < 0.01) due to the need for the players to sprint as fast as possible to score (attacker) or prevent the scoring of a goal (defender).

Our study showed that TGs were effective ways to achieve large distances covered at high speed (Table 1). Since the 1 vs. 1_Behind_ format allowed players to reach a greater PeakV, it also showed greater DC ≥ 24 km•h^−1^ in comparison with other tasks (ES > 1.8; *p* < 0.001) (Figure1). Therefore, the 1 vs. 1_Behind_ format resulted in the greatest distances covered at high speeds, while 1 vs. 0 and 1 vs. 1_Front_ formats led to more distance covered at the speed band of 18–23.9 km•h^−1^ (Figure 1). Those TGs in which players in possession of the ball had no opposition between them and the goal (1 vs. 0 and 1 vs. 1_Behind_) performed more high-speed actions. However, when the ball possessor encountered an opponent on his way to the goal (1 vs. 1_Front_), the high-speed distances were reduced. This may be due to the fact that these tasks require faster actions due to the lack of execution time to exploit the competitive advantage and to the characteristics of decision making in a dribbling or a non-dribbling situation. In line with our results, previous studies have also shown alterations in high velocity in TGs as a result of modifications in the duration of the sets, field dimensions, and players’ ratios (Asian-Clemente et al., 2023a, 2023b). Our results showed 77.59–103.73 DC 18–20.9 km•h^−1^ (m), 98.6–172.59 DC 21–23.9 km•h^−1^ (m) and 117.93–351.72 DC ≥ 24 km•h^−1^ (m) in the three TGs analyzed. These results are superior to the data provided for high-intensity running and sprint distance in other task formats. For example, Asian-Clemente at al. (2022), showed in large-sided games (60 x 60 m) 48.8 DC 18–21 km•h^−1^ (m) and 28.1 DC > 21 km•h^−1^ (m), in SSGs with change of the zone (60 x 40 m) 118.4–84.2 (m) and TGs (60 x 40 m) 201.5–255.9 (m). These tasks were organized in three sets of 4-min duration with 2 min of recovery, in contrast to our study with two sets of 8 min.

The results of our study showed a high accelerative component during TGs when attackers could freely attack the goal without the opposition or when the defender was behind (ES > 3.03; *p* < 0.001), with no significant differences between them (ES = 0.12; *p* > 0.05). For decelerations, the TGs carried out showed a low number in comparison with the number of accelerations (Table 1), being highest for the 1 vs. 1_Front_ format (ES > 0.81; *p* < 0.01). The acceleration and deceleration outcomes could be related to the duration of the actions and the rest-work ratio distribution. Greater intermittency could be expected in 1 vs. 1_Behind_ and 1 vs. 0 formats compared to the 1 vs. 1_Front_ format because of their longer duration due to the attacker-defender interaction. Our results are consistent with previous research carried out in reduced space in soccer training (Asín-Izquierdo et al., 2023; Fanchini et al., 2011; Köklü et al., 2017), where activities with higher intermittency indicated greater external loads, possibly related to differences in the task context (as in the previous paragraph with space-time analysis, presence of an adversary and decision making). TGs and small-sided games can complement each other, as small-sided games involve high accelerations and decelerations with low demands for high speed, while TGs feature low accelerations and decelerations with high demands for high speed. The results obtained are in line with previous studies using TGs in terms of accelerations and decelerations, which found differences between particular formats (Asian-Clemente et al., 2023a, 2023b). The results of our study provide values for accelerations and decelerations of 6.23–15.21 and 1.70–6.43, respectively, which were similar ranges to those found in previous studies when manipulations of space (6.1–10.1 and 4.9–7.4) and time (3.9–12.3 and 7.7–14.5) were evaluated in TGs (Asian-Clemente et al., 2023a, 2023b).

In a similar fashion, because the three TGs displayed different locomotor and mechanical values, internal loads behaved accordingly depending on the TG (Table 1). In the absence of a defender who could increase the complexity and difficulty of the task, the 1 vs. 0 format showed the lowest RPE scores (ES > 0.82; *p* < 0.01). It has been demonstrated that cognitively demanding tasks can also increase RPE values (Inoue et al., 2022; Macpherson et al., 2019). Our study found RPEs ranging from 6.11 to 7.13, which is a different range to that previously found when space manipulation (Asian-Clemente et al., 2023a) was studied. Asian-Clemente et al. (2023a) showed RPEs ranging from 4.3 to 6.4, with higher values being recorded as the space increased. On the other hand, both studies obtained lower values than those observed in a TG time study (Asian-Clemente et al., 2023b), which indicated values ranging from 9.2 to 9.8. These discrepancies may be because the duration of TGs in the latter case was significantly higher (between 15 and 60 s).

Finally, the tasks analyzed showed that fatigue caused significant reductions in sprint and jump values. Despite the fact that there were no differences between particular tasks, the clearest differences were found in the 1 vs. 1_Front_ format, which was perhaps related to the greater neuromuscular fatigue induced by direct attacker-opponent interaction. This should also be understood as a greater frequency of rest intervals within the sets accompanied by a decrease in neuromuscular fatigue and metabolic stress associated with different factors, highlighting the pace in this type of sport (Ferraz et al., 2018; Girard et al., 2011; Mendez-Villanueva et al., 2012). These results related to the demands of this type of a task concur with previous evidence of deterioration in sprint and CMJ performance after matches, other specific tasks (Silva et al., 2018; Sparkes et al., 2018, 2020) and TGs (Asian-Clemente et al., 2023b).

The main limitations of this study were the small sample of participants located in only one homogeneous group of soccer players with a similar level of performance. It would be interesting to analyse players with different performance levels and teams from different locations, which would allow for a broader sample of similar players. On the other hand, although the total time of the tasks was similar and all participants performed the same number of repetitions, each task may have a different effective time for its execution. Future studies should differentiate between the roles according to the phase of the game (attack-defense), comparing the demands in this type of a task.

## Conclusions

The presence or absence of an opponent and their context of participation in the action modulates the conditional response of soccer players. These constraints affect the internal and external demands of TGs. The three analyzed TGs showed high peak velocity data. The TGs with an opponent behind and without an opponent showed higher speeds, sprints and accelerations than exercises with an opponent in front, which led to improved decelerations. The internal load evaluated using the RPE was higher in exercises with an opponent. The three tasks seemed to induce fatigue, as reflected in the decrease in sprint time and CMJ performance, with the opponent in front format showing the most marked changes. Taking into account these aspects, this study affirmed that external and internal loads in TGs are affected by the presence of opponents and by their initial position.

## Practical Implications

The results obtained may help coaches and sport scientists understand the orientation of the load of TGs, depending on the presence or absence of opponents and their context at the beginning of the intervention. TGs offer flexibility to coaches, as these tasks are suitable for preparing players for competition and allow training for both injured and non-starting players. These tasks can be introduced during central moments of a training week with high specificity, favoring the transfer to the learning of technical-tactical principles, while at the same time reproducing the demands of competition in terms of high intensity, speed, and generated fatigue. In addition, these TGs could be used in situations when there are few participants, and for their usefulness in compensation sessions with substitutes (the day after the match) or players undergoing recovery periods, when the training load of these players could be modified according to the desired objective. Introducing these games in the complements or in MD + 1 for non-starting players may be an appropriate strategy to reproduce high-demanding situations specific for soccer.

## Data sharing statement

Data are available upon reasonable request.
